# Gene expression prognostic of early relapse risk in low‐risk B‐cell acute lymphoblastic leukaemia in children

**DOI:** 10.1002/jha2.872

**Published:** 2024-03-15

**Authors:** Xiaowen Gong, Tianyuan Hu, Qiujin Shen, Luyang Zhang, Wei Zhang, Xueou Liu, Suyu Zong, Xiaoyun Li, Tiantian Wang, Wen Yan, Yu Hu, Xiaoli Chen, Jiarui Zheng, Aoli Zhang, Junxia Wang, Yahui Feng, Chengwen Li, Jiao Ma, Xin Gao, Zhen Song, Yingchi Zhang, Robert Peter Gale, Xiaofan Zhu, Junren Chen

**Affiliations:** ^1^ State Key Laboratory of Experimental Hematology National Clinical Research Center for Blood Diseases Haihe Laboratory of Cell Ecosystem Institute of Hematology and Blood Diseases Hospital Chinese Academy of Medical Sciences and Peking Union Medical College Tianjin China; ^2^ Tianjin Institutes of Health Science Tianjin China; ^3^ Department of Immunology and Inflammation Centre for Haematology Imperial College of Science Technology and Medicine London UK

**Keywords:** acute lymphoblastic leukaemia, children, *ETV6::RUNX1*, leukaemia relapse, measurable residual disease

## Abstract

*ETV6*::*RUNX1* is the most common fusion gene in childhood acute lymphoblastic leukaemia (ALL) and is associated with favorable outcomes, especially in low‐risk children. However, as many as 10% of children relapse within 3 years, and such early relapses have poor survival. Identifying children at risk for early relapse is an important challenge. We interrogated data from 87 children with low‐risk *ETV6*::*RUNX1*‐positive B‐cell ALL and with available preserved bone marrow samples (discovery cohort). We profiled somatic point mutations in a panel of 559 genes and genome‐wide transcriptome and single‐nucleotide variants. We found high *TIMD4* expression (> 85th‐percentile value) at diagnosis was the most important independent prognostic factor of early relapse (hazard ratio [HR] = 5.07 [1.76, 14.62]; *p* = 0.03). In an independent validation cohort of low‐risk *ETV6*::*RUNX1*‐positive B‐cell ALL (*N* = 68) high *TIMD4* expression at diagnosis had an HR = 4.78 [1.07, 21.36] (*p* = 0.04) for early relapse. In another validation cohort including 78 children with low‐risk *ETV6*::*RUNX1*‐negative B‐cell ALL, high *TIMD4* expression at diagnosis had an HR = 3.93 [1.31, 11.79] (*p* = 0.01). Our results suggest high *TIMD4* expression at diagnosis in low‐risk B‐cell ALL in children might be associated with high risk for early relapse.

## 1 INTRODUCTION

In childhood acute lymphoblastic leukaemia (ALL), ‘early relapse’ is often defined as ‘< 3 years after diagnosis’ and is more challenging to salvage compared to late relapse [1, 2]. After re‐induction, these children may receive a haematopoietic cell transplant, but survival remains poor [2]. Early and late relapses have different gene expression patterns and are two distinct types of disease progression [3, 4].

*ETV6::RUNX1* is the most common fusion gene in childhood B‐cell ALL [5]. Although associated with a lower risk of relapse, as many as 10% of children with *ETV6::RUNX1*‐positive B‐cell ALL relapse within 3 years after therapy starts [6, 7, 8, 9]. Prior studies reported molecular abnormalities associated with relapse in childhood ALL, but they did not identify a molecular signature that is present at diagnosis and is prognostic of early relapse in *ETV6::RUNX1*‐positive B‐cell ALL [4, 10–22]. We conducted a multi‐omics analysis including genomic variations (targeted sequencing of 559 genes and genome‐wide single‐nucleotide variants [SNVs]) and transcriptome in 87 consecutive children (age ≤10) with low‐risk *ETV6::RUNX1*‐positive B‐cell ALL. We identified high *TIMD4* expression at diagnosis as a prognostic factor for early relapse risk and validated our finding in an independent cohort of 68 children with low‐risk *ETV6::RUNX1*‐positive B‐cell ALL and another independent cohort of 78 children with low‐risk *ETV6::RUNX1*‐negative B‐cell ALL.

## 2 MATERIALS AND METHODS

### 2.1 Definition of low risk at diagnosis

*ETV6::RUNX1*‐positive B‐cell ALL subjects were classified as *low‐risk at diagnosis* if they lacked hypo‐diploidy (< 44 chromosomes), t(1;19), t(9;22), *KMT2A* rearrangement or intra‐chromosomal amplification of chromosome 21 (iAMP21) and did not have central nervous system (CNS) 3 state (white blood cell [WBC] ≥ 5 × 10E+6/L with blasts in cerebrospinal fluid [CSF]) or testes leukaemia at diagnosis. For *ETV6::RUNX1*‐negative B‐cell ALL subjects, *low‐risk at diagnosis* was defined similarly but with the addition requirement that age was between 1 and 10 with WBC < 50 × 10E+9/L.

A note on the inclusion of initial WBC count in risk‐stratification criteria: The National Cancer Institute (NCI) standard risk is defined as age between 1 and 10 with WBC < 50 × 10E+9/L [23]. WBC count, however, is reportedly not a prognostic factor in *ETV6::RUNX1*‐positive childhood ALL when treated on contemporary, more modern protocols [8]. In contrast, WBC count is prognostic in subjects with hyper‐diploidy [24]. Accordingly, initial WBC count was not included as a criterion for low‐risk in *ETV6::RUNX1*‐positive childhood ALL in Total Therapy 16 and CCCG‐ALL‐2015 protocols [25, 26].

### 2.2 Discovery cohort

**FIGURE 1:**
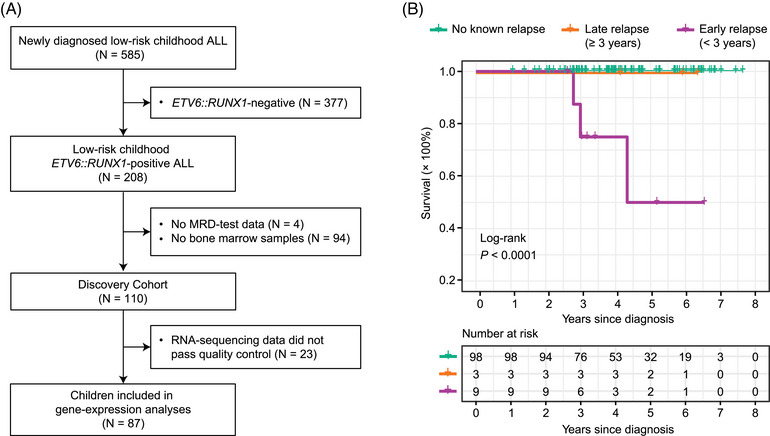
Discovery Cohort. (A) Flow chart for patient selection. (B) Survival: early relapses vs. late relapses vs. other cases.

This study was approved by the Academic Committee (IIT‐NI2020001) and Ethics Review Committee (NI2020001‐EC‐1) of the Institute of Hematology, Chinese Academy of Medical Sciences (IHCAMS). We interrogated data from 110 children with low‐risk *ETV6::RUNX1*‐positive B‐cell ALL treated on the CCCG‐ALL‐2015 protocol from July 2015 to September 2020 who had available preserved bone marrow samples (Figure 1A and Table 1) [26]. Subjects and/or guardians gave written informed consent consistent with the precepts of the Helsinki Declaration. Relapse was defined as (1) bone marrow blasts ≥5%, (2) isolated extra‐medullary relapse in CSF or testes or (3) a positive measurable residual disease (MRD)‐test in histological complete remission. The last follow‐up was on May 15, 2023.

**TABLE 1:** Clinical characteristics of children in the discovery and validation cohorts.

	Discovery Cohort	Validation Cohort #1	Validation Cohort #2	
	*ETV6::RUNX1*‐positive China (*N* = 110)	*ETV6::RUNX1*‐positive US (*N* = 68)	*ETV6::RUNX1‐*negative US (*N* = 78)	*p*‐Value
**Age at diagnosis, median years (range)**	4.0 (1.4–10.0)	3.8 (1.7–9.9)	3.7 (1.4–9.8)	0.47^*^
**Biological sex, *N* (%)**				0.59^†^
Male	57 (51.8)	40 (59)	40 (51)	
Female	53 (48.2)	28 (41)	38 (49)	
**Ethnicity, *N* (%)**				<0.001^†^
White	0 (0.0)	51 (75)	62 (79)	
Black	0 (0.0)	10 (15)	6 (8)	
Asian	110 (100.0)	0 (0)	1 (1)	
Black or African American	0 (0.0)	2 (3)	1 (1)	
Other or Unknown	0 (0.0)	5 (7)	8 (10)	
**WBC count at diagnosis, median** **× 10E+9/L (range)**	6.9 (0.9–93.1)	23.8 (2.1–117.6)	13.0 (0.2–284.6)	<0.001^*^
**Disease risk at diagnosis, *N* (%)** ^a^				–
Low‐risk	110 (100.0)	68 (100)	78 (100)	
**Treatment regimen, *N* (%)**				<0.001^†^
CCCG‐ALL‐2015	110 (100.0)	–	–	
AALL0232	–	14 (21)	–	
AALL0331	–	7 (10)	26 (33)	
Total Therapy 16	–	47 (69)	52 (67)	
**MRD ≥ 1 × 10E–2 on day 15/19, *N* (%)**	11 (10.0)	3 (6)^b^	19 (37)^c^	<0.001^†^
**MRD > 0 on day 46, *N* (%)**	23 (20.9)	8 (18)^d^	5 (10)^e^	<0.001^†^
**Follow‐up duration, median years (IQR)**	4.1 (3.0–5.7)	9.6 (6.6–10.2)	9.6 (5.7 –10.1)	<0.001^§^
**Cumulative incidence of relapse, % (95% CI)**				0.002^‡^
3 years	9.2 (4.5, 16.0)	10.6 (4.6, 19.4)	17.9 (10.3, 27.2)	
5 years	15.2 (7.9, 24.8)	13.6 (6.7, 23.1)	33.3 (23.1, 43.9)	
**Survival, % (95% CI)**				0.018^§^
3 years	97.8 (94.8, 100.0)	95.5 (90.7, 100.0)	93.6 (88.3, 99.2)	
5 years	95.7 (90.9, 100.0)	92.5 (86.4, 99.0)	85.9 (78.5, 94.0)	

Abbreviations: ALL, acute lymphoblastic leukaemia; CI, confidence interval; IQR, interquartile range; MRD, measurable residual disease; WBC, white blood cell.

^a^ Risk‐stratification was based on subject characteristics *at diagnosis* according to CCCG‐ALL‐2015 criteria.

^b^ 47 subjects had data.

^c^ 52 subjects had data.

^d^ 45 subjects had data.

^e^ 51 subjects had data.

* Kruskal‐Wallis test.

^†^ Chi‐squared test.

^‡^ Gray test.

^§^ Two‐tailed log‐rank test.

### 2.3 Bone marrow samples

Mononuclear cells were isolated by density‐gradient centrifugation using Ficoll‐Paque media (TBDscience), viably frozen in fetal bovine serum (Gibco) with 10% dimethyl sulfoxide (Sigma‐Aldrich) and stored at –196°C. Bulk sequencing was conducted on the isolated mononuclear cells (more details are provided below).

### 2.4 Detection of somatic point mutations

We did next‐generation sequencing of 559 genes to identify somatic point mutations (Table S1).

Genomic DNA was extracted from bone marrow mononuclear cells using the Universal Genomic DNA Kit (CoWin Biosciences) according to the manufacturer's instructions and quantified by agarose gel electrophoresis and Qubit 2.0 Fluorometer (Life Technologies). Genomic DNA was ≥ 0.1 µg per sample. DNA was fragmented by sonication to a size range of 180–280 bp. Fragmented DNA was end‐polished, A‐tailed and ligated with adapters to generate DNA libraries using NEB Next Ultra DNA Library Prep Kit (New England Biolabs). Exon‐containing fragments were captured, proliferated and purified using Agilent SureSelect XT Custom Kit (Agilent Technologies) and then quantified with Agilent Bioanalyzer 2100 (Agilent Technologies). Paired‐end sequencing was performed on Illumina NovaSeq 6000 (Illumina). The average sequencing depth was 832× (range 510–1604).

The quality of fastq files was checked by FastQC (v.0.11.4, http://www.bioinformatics.bbsrc.ac.uk/projects/fastqc). Sequencing artefacts and low‐quality reads were discarded using Trimmomatic (v.0.35) [27]. Filtered reads were aligned to the reference human genome (hg19) using Burrows‐Wheeler Aligner (BWA, v.0.7.15‐r1140) [28]. SAMtools (v.1.3.1) was used for bam file indexing [29]. Variants including SNV, short insertion and deletion were called by Genome Analysis Toolkit (GATK, v.4.1.4.1), using matched samples collected in post‐induction remission as a proxy for germline genotype [30]. The Ensembl Variant Effect Predictor (VEP, v.96) was then used to annotate each called variant [31].

Somatic point mutations were defined as (1) mutation detected in > 2 out of > 10 reads; (2) non‐synonymous mutations; (3) mutations within exons or at splicing sites; (4) variant allele frequency (VAF) 0.01–0.98 and (5) < 0.01 frequency in ExAC and gnomAD databases.

### 2.5 Transcriptome sequencing and gene‐expression analyses

Bone marrow‐derived mononuclear cells collected at diagnosis were lysed in TRIzol reagent (Invitrogen) and total RNA was extracted using the RNeasy MiniElute Cleanup Kit (QIAGEN, Hilden, Germany) according to the manufacturer's instructions. The RNA‐sequencing library was constructed using the NEBNext Ultra RNA Library Prep Kit for Illumina (New England Biolabs). Sequencing was performed on Illumina NovaSeq 6000 generating 150 bp paired‐end reads, which were then aligned to the reference human genome hg19 using STAR (v2.6.1d) [32]. For each sample, the results of RNA sequencing were deemed usable only if at least half of the reads were mapped to opening reading frames. The expression level of a gene was calculated as ‘fragments per kilobase of transcript per million fragments mapped’ (FPKM).

Fusion transcripts were detected by STAR‐Fusion [33] after STAR analysis and then filtered by excluding: (1) mappings to repetitive regions; (2) fusions of non‐protein‐coding genes; (3) mappings outside of reading frames; (4) junctions not at exon‐intron boundaries; (5) fusions between homologs; (6) < 2 pairs of breakpoint‐spanning reads or < 2 reads covering the breakpoint; and (7) frame‐shifts.

### 2.6 Genome‐wide SNV analyses

Genomic DNA from bone marrow samples was genotyped using the Infinium Human Asian Screening Array‐24 v1.0 (Illumina) gene chip, which covers 750,000 genomic loci. Genotyping read‐outs were analyzed by GenomeStudio software (Illumina). All samples passed the quality control (call rate > 95%). SNV calls were excluded if Illumina GenTrain quality score was < 0.8 or minor allele frequency was ≤ 5%. We focused on SNVs located in autosomes. A final set of 335,206 SNVs were identified. Genotypes (AA, AB and BB) of individual SNVs were first converted to numbers (0, 1 and 2) and then normalized and analyzed using principal component analysis as described [34].

### 2.7 Measurable residual disease

All subjects in Discovery Cohort had MRD‐test data 19 and 46 days after therapy started [35]. MRD was quantified by multi‐parameter flow cytometry (MPFC; BD FACSCanto Plus flow cytometer [V657338000225; BD Biosciences] with BD FACSDiva Software v8.0.1) analyses of bone marrow samples using antibodies to CD19 (PE‐Cy7 IM3628; Beckman Coulter, Brea, CA, USA), CD10 (PE A07760; Beckman Coulter), CD34 (PerCP‐Cy5.5 343522; BioLegend), CD20 (APC‐H7 641396; BD Biosciences), CD22 (BV421 302524; BioLegend), CD38 (FITC A07778; Beckman Coulter), CD45 (V500 662912; BD Biosciences) and CD81 (APC 551112; BD Biosciences). Leukaemia cells were defined by either leukaemia‐associated immune phenotype (LAIP) identified at diagnosis or an immune phenotype deviating from normal haematopoietic cells [36]. ≥ 1 × 10E–2 detectable residual cancer cells in a bone marrow sample collected on day 19 was defined as positive MRD. Any detectable residual cancer cell in a bone marrow sample collected on day 46 was defined as positive MRD.

### 2.8 Validation cohorts

To validate the effect of *TIMD4* expression level on early‐relapse risk, we interrogated a cohort of 68 children with low‐risk *ETV6::RUNX1*‐positive B‐cell ALL (‘Validation Cohort #1′; Table 1). Clinical data of 21 cases treated on Children's Oncology Group (COG) AALL0232 (*N* = 14) and AALL0331 (*N* = 7) were acquired from the publicly accessible database ‘Therapeutically Applicable Research to Generate Effective Treatments (TARGET) ALL Phase‐2′ (https://portal.gdc.cancer.gov/projects/TARGET‐ALL‐P2) [37]. Clinical data of 47 cases treated on Total Therapy 16 were acquired from Jeha et al. [38]. All the subjects in Validation Cohort #1 were low‐risk at diagnosis according to the CCCG‐ALL‐2015 or Total Therapy 16 criteria. The 14 subjects treated on COG AALL0232 had WBC > 50 × 10E+9/L (median = 78.6 × 10E+9/L; range, 51.6–117.6 × 10E+9/L), a factor that is reportedly not prognostic in *ETV6::RUNX1*‐positive childhood ALL treated on modern protocols [8]. The other 54 subjects met both the age and WBC criteria for NCI standard risk [23]. Forty‐five (66%) subjects had MRD‐test data on day 46, of which eight (18%) had positive MRD. The estimated positive‐MRD rates on day 46 were statistically indistinguishable between Discovery Cohort and Validation Cohort #1 (*p* = 0.82).

To further investigate if *TIMD4* expression has similar effect on relapse risk when there is no *ETV6::RUNX1* fusion gene, we used a cohort of 78 children with low‐risk (based on subject characteristics *at diagnosis* according to CCCG‐ALL‐2015 criteria) *ETV6::RUNX1*‐negative B‐cell ALL (‘Validation Cohort #2′; Table 1), of which 26 were treated on COG AALL0331 and 52 on Total Therapy 16 [37, 38]. All the subjects in Validation Cohort #2 were low‐risk at diagnosis according to the CCCG‐ALL‐2015 or Total Therapy 16 criteria, and all were NCI standard‐risk. Fifty‐one (65%) subjects had MRD‐test data on day 46, of which five (10%) had positive MRD. The estimated positive‐MRD rates on day 46 were statistically indistinguishable between Discovery Cohort and Validation Cohort #2 (*p* = 0.13).

Gene expression data were downloaded from https://viz.stjude.cloud/st‐jude‐childrens‐research‐hospital/visualization/pax5‐driven‐subtypes‐of‐b‐progenitor‐acute‐lymphoblastic‐leukemia‐genomepaint~16.

### 2.9 Determination of cut‐off value for *TIMD4* expression level

The lowest cut‐off percentile value for classifying *TIMD4* expression into a binary (high vs. low) with *p* < 0.05 were identified using Discovery Cohort. This ideal cut‐off value was then applied to the validation cohorts.

### 2.10 Statistics

Statistical analyses were done in the R computing language. Single‐ and multi‐variable relapse risk analyses used the Cox proportional hazards model, treating non‐relapse mortality as right‐censoring. Multiple testing was adjusted by the Bonferroni method [39]. The false discovery rate (FDR) was set to be < 0.1 when conducting initial screenings on point mutations in the sequenced gene panel and genome‐wide gene expression levels. Otherwise, (adjusted) *p* < 0.05 was considered statistically significant. To exclude the possibility our conclusion was a false positive occurring purely by chance, we also performed permutation analyses on Discovery Cohort by scrambling gene expression levels of the children for whom RNA‐sequencing data were available.

Concordance (‘C‐statistic’) between raw *TIMD4* expression value (measured in FPKM) and early relapse time was calculated using the ‘coxph’ function in R.

## 3 RESULTS

From July 2015 to September 2020, there were 208 children (age ≤10) with low‐risk *ETV6::RUNX1*‐positive ALL treated on CCCG‐ALL‐2015 at the IHCAMS. One hundred and ten subjects who had available preserved bone marrow samples were used as Discovery Cohort (Figure 1A). Discovery Cohort's subject co‐variates are displayed in Table 1. There was no significant difference between Discovery Cohort and the other *ETV6::RUNX1*‐positive ALL cases that had no preserved bone marrow samples (Table S2). Note, however, that early‐relapse risk was 9% in subjects with available bone marrow samples and 4% in subjects without bone marrow samples (*p* = 0.16; Table S2). This discrepancy could be caused by attendant biases when physicians decided whose bone marrow samples to preserve.

Fifty‐seven (52%) of Discovery Cohort were male. Median age was 4 years (interquartile range [IQR], 3.2–5.6 years). The median WBC at diagnosis was 6.9 × 10E+9/L (IQR, 4.3–15.2 × 10E+9/L). Median follow‐up was 4.1 years (IQR, 3.0–5.7 years). *ETV6::RUNX1* was detected using fluorescence in‐situ hybridization (FISH; *N* = 3), quantitative reverse‐transcription polymerase chain reaction (RT‐qPCR; *N* = 8) or both (*N* = 99).

All subjects in Discovery Cohort achieved a histological complete remission. There were nine early relapses, of which two were isolated testes relapses. Three‐year cumulative incidence of relapse (CIR) was 9% (95% confidence interval [CI], 5, 16%). Five early relapses including one isolated testis relapse were in children with a negative MRD test on day 46 (during early consolidation chemotherapy). Four of 23 children with a positive MRD test on day 46 had early relapses. Put otherwise, the positive predictive value of MRD testing on day 46 for early relapse was 17% and the false‐negative rate was 6%.

Three‐ and five‐year survival rates of Discovery Cohort were 98% and 96%, respectively (Table 1). Children with early relapses had significantly worse survival than the other children (*p* < 0.0001; Figure 1B). Unlike early relapses, most of the late relapses were salvageable as indicated by their 5‐year survival rate of 100% (Figure 1B).

Forty‐seven children in Discovery Cohort (43%) had complete or partial deletion of the non‐translocated copy of *ETV6* in 21–95% (median 70%) of *ETV6::RUNX1*‐positive cells at diagnosis. *ETV6* deletion was not correlated with the risk of early relapse (HR = 1.06 [0.28, 3.95]; *p* = 0.93).

**FIGURE 2:**
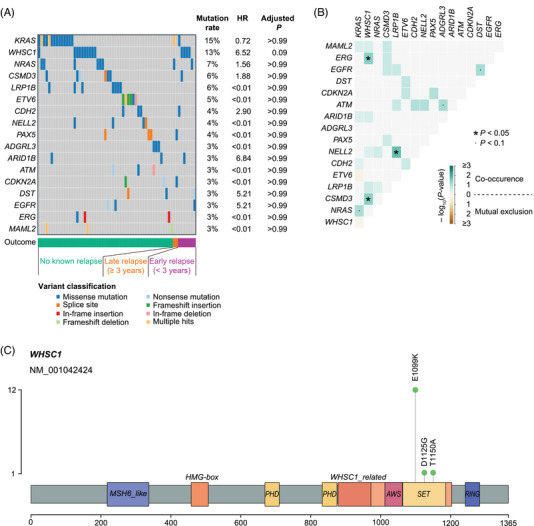
Spectra of point mutations in Discovery Cohort. (A) Recurrent point mutations (mutation rate ≥ 2%). (B) Co‐occurrence and mutual exclusion of point mutations. *p‐*values were based on pair‐wise Fisher's exact test. (C) *WHSC1* point mutations.

All bone marrow samples collected at diagnosis in Discovery Cohort had blast percentage > 50% (median = 75%; Interquartile Range [IQR], 66.1%–85.6%). The most common somatic point mutations in Discovery Cohort were in *KRAS* (*N* = 16), *WHSC1* (*N* = 14), *NRAS* (*N* = 8), *CSMD3* (*N* = 7), *LRP1B* (*N* = 7) and *ETV6* (*N* = 6; Figure 2A). Point mutations in *WHSC1* were associated with co‐mutations in *ERG* and *CSMD3* (Figure 2B). Also, point mutations in *NELL2* and *LRP1B* were correlated. There was no other significant co‐occurrence or mutual exclusion of point mutations. Only point mutation in *WHSC1* (HR = 6.52 [1.75, > 10]; adjusted *p* = 0.09) correlated with early relapse risk after controlling the FDR at < 0.1 (Figure 2A). In 12 children with *WHSC1* point mutation, the mutation was E1099K (Figure 2C).

Neither of the first two principal components of whole‐genome SNVs was correlated with early relapse (*p* > 0.05).

Twenty‐three children's RNA‐sequencing results did not meet the criteria for quality control (**Methods**; Figure 1A). We were able to collect RNA‐sequencing data on 87 children, seven of whom had early relapses. Using these data we identified 14 additional in‐frame fusion genes in 12 cases (Table 2). Having ≥ 1 additional fusion gene was not associated with early relapse (HR = 2.45 [0.48, 12.62]; *p* = 0.28).

**TABLE 2:** Additional in‐frame fusion genes detected by RNA‐sequencing in Discovery Cohort.

Patient ID	Left breakpoint			Right breakpoint			Fusion gene
	Chromosome	Position	Direction	Chromosome	Position	Direction	
TEL_23	6	87865477	+	6	89793472	+	*ZNF292::PNRC1*
TEL_25	1	78432568	–	19	10465285	–	*FUBP1::TYK2*
TEL_25	1	245026976	–	1	7792508	+	*HNRNPU::CAMTA1*
TEL_27	5	180076488	–	2	242644068	+	*FLT4::ING5*
TEL_43	3	142166712	–	3	142168444	–	*XRN1::ATR* ^*^
TEL_43	13	41556119	–	13	41134997	–	*ELF1::FOXO1* ^*^
TEL_51	11	128564171	+	3	111812207	+	*FLI1::C3orf52*
TEL_53	21	36421139	–	12	16185476	+	*RUNX1::DERA*
TEL_64	2	198175302	–	2	136873482	–	*ANKRD44::CXCR4*
TEL_86	11	43380663	+	11	46693805	+	*TTC17::ATG13*
TEL_129	10	97810223	+	10	99327671	+	*CCNJ::UBTD1*
TEL_155	17	67411141	+	22	32352126	+	*MAP2K6::YWHAH*
TEL_163	17	80223562	–	9	131453449	+	*CSNK1D::SET*
TEL_227	3	49363152	–	2	7001516	–	*USP4::CMPK2*

Asterisks denote fusion genes that have been previously reported.

**FIGURE 3:**
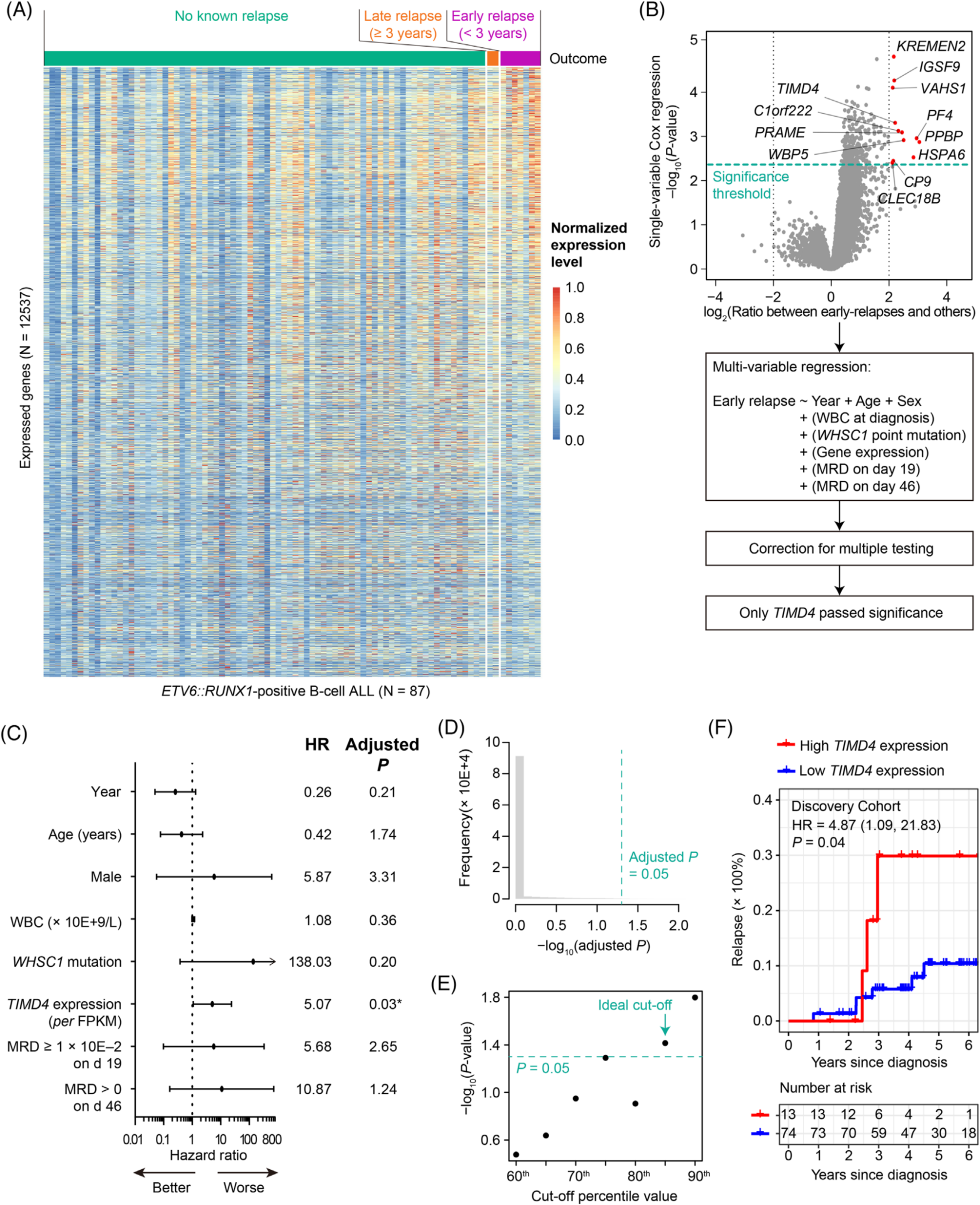
*TIMD4* expression level at diagnosis is an independent prognostic factor for early relapse in Discovery Cohort. (A) Gene expression levels. (B) Analytic pipeline for confirming gene expression level's effect on early relapse risk using multi‐variable analysis. (C) Cox model prognosing early relapse. (D) Distribution of adjusted *p‐*value for *TIMD4* expression's effect on early relapse risk in permutation experiments (1 × 10E+5 trials). (E) Identifying the ideal cut‐off value for *TIMD4* expression level. (F) Cumulative incidence of relapse in *ETV6::RUNX1*‐positive B‐cell ALL: high vs. low *TIMD4* expression at diagnosis.

Gene expression patterns at diagnosis correlated with early relapse (Figure 3A). Expression level (measured in FPKM) of 12 genes (*KREMEN2*, *IGSF9*, *VASH1*, *TIMD4*, *C1orf222*, *PRAME*, *PF4*, *WBP5*, *PPBP*, *HSPA6*, *GP9* and *CLEC18B*) at diagnosis was significantly associated with early relapse (FDR < 0.1; Figure 3B). To isolate a gene or genes that provide independent prognostic power for early relapse risk, we performed multivariable analyses including year, sex, age, WBC at diagnosis, *WHSC1* point mutation, MRD on day 19 and MRD on day 46 (Figure 3B). Out of the 12 candidate genes, only *TIMD4*’s expression level was independently associated with early relapse (HR = 5.07 [1.76, 14.62] per FPKM; *p* = 0.0026; adjusted *p* = 0.03; Figure 3C).

To assess the possibility that *TIMD4* was a chance false‐positive hit, we conducted permutation analyses on Discovery Cohort. Based on 1 × 10E+5 trials of in silico experiments in which we scrambled *TIMD4* expression level values of the 87 children for whom RNA‐sequencing data were available (Figure 3D), we found FDR to be 1.4 × 10E–4; that is, only 14 out of 1 × 10E+5 trials of permutation experiments falsely identified *TIMD4* expression as associated with early relapse.

In Discovery Cohort, the C‐statistic between raw, quantitative *TIMD4* expression level at diagnosis and early relapse was 0.68 (standard error [se] = 0.10). 85th‐percentile value was the lowest cut‐off percentile value for classifying *TIMD4* expression into a binary (high vs. low) with *p* < 0.05 (Figure 3E). Children with high (top 15%) *TIMD4* expression at diagnosis had an HR = 4.87 [1.09, 21.83] (*p* = 0.04) for early relapse compared to the other children (Figure 3F).

We also conducted a discordance analysis between MRD‐testing and *TIMD4* expression (Table 3). Three early relapses in Discovery Cohort were preceded by high *TIMD4* expression at diagnosis without a positive MRD test on day 46, while three were preceded by a positive MRD test on day 46 without high *TIMD4* expression at diagnosis. One escaped early detection despite both MRD‐testing during early consolidation and measurement of *TIMD4* expression level at diagnosis. Therefore, in several children with early relapses, the results of *TIMD4*‐expression‐testing were more accurate compared with MRD‐testing in prognosing relapse.

**TABLE 3:** Discordance analysis in Discovery Cohort: *TIMD4* expression at diagnosis vs. measurable residual disease (MRD) on day 46 (during early consolidation).

		*TIMD4* expression at diagnosis	
		High (top 15%)	Low (bottom 85%)
**MRD on day 46**	> 0	0	3
	= 0	3	1

Numbers in the cells indicate the number of early relapses.

**FIGURE 4:**
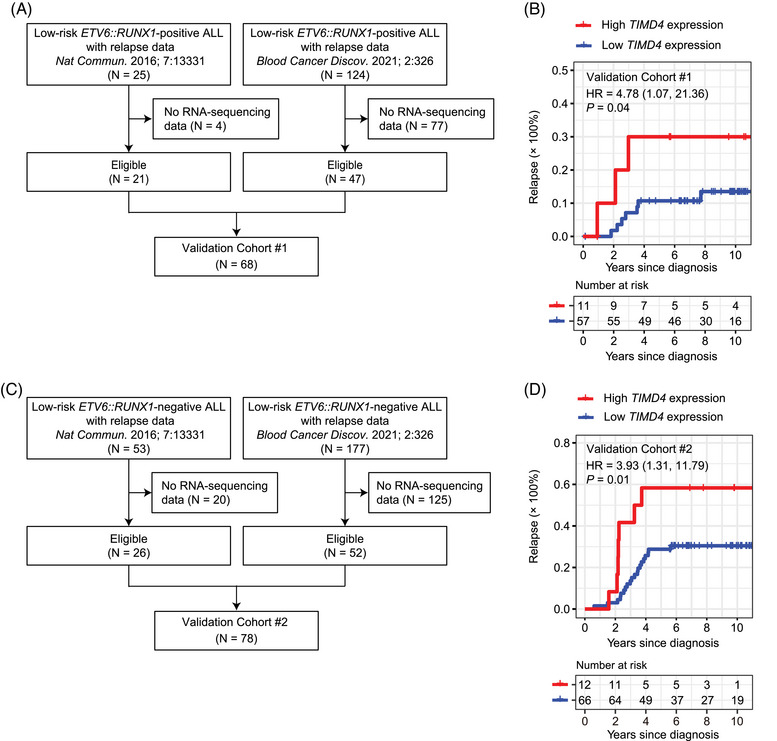
Effect of *TIMD4* expression level at diagnosis on early relapse in the validation cohorts. (A) Patient selection flow chart for Validation Cohort #1: low‐risk *ETV6::RUNX1*‐positive B‐cell ALL. (B) Cumulative incidence of relapse in Validation Cohort #1: high vs. low *TIMD4* expression at diagnosis. (C) Patient selection flow chart for Validation Cohort #2: low‐risk *ETV6::RUNX1*‐negative B‐cell ALL. (D) Cumulative incidence of relapse in Validation Cohort #2: high vs. low *TIMD4* expression at diagnosis.

Because there were potential ascertainment biases in Discovery Cohort, it is crucial to validate our finding in an independent cohort of children with low‐risk *ETV6::RUNX1*‐positive B‐cell ALL. We interrogated a cohort of 68 children from the US (Validation Cohort #1; Figure 4A; Table S3). Subject co‐variates at diagnosis in Validation Cohort #1 are displayed in Table 1. There were seven early relapses. Three‐year CIR was 11% (5, 19%), comparable to Discovery Cohort (*p* = 0.97). The C‐statistic between raw, quantitative *TIMD4* expression level at diagnosis and early relapse was 0.74 (se = 0.09). Children with high (top 15%) *TIMD4* expression at diagnosis had an early‐relapse HR = 4.78 [1.07, 21.36] (*p* = 0.04; Figure 4B).

Finally, to explore if the association between *TIMD4* expression at diagnosis and CIR might extend to children without *ETV6::RUNX1*, we also interrogated a cohort of 78 children with low‐risk *ETV6::RUNX1*‐negative B‐cell ALL from the US (Validation Cohort #2; Figure 4C; Table S4). Subject co‐variates at diagnosis in Validation Cohort #2 are displayed in Table 1. There were 14 early relapses. Three‐year CIR was 17.9% (10.3, 27.2%) and worse than Discovery Cohort, but the difference between Discovery Cohort and Validation Cohort #2 did not pass statistical significance (*p* = 0.07). The C‐statistic between raw, quantitative *TIMD4* expression level at diagnosis and early relapse was 0.62 (se = 0.06). Children with high (top 15%) *TIMD4* expression at diagnosis had an early‐relapse HR = 3.93 [1.31, 11.79] (*p* = 0.01; Figure 4D).

Altogether, the current study included 13 high‐expressers of *TIMD4* in Discovery Cohort, 11 in Validation Cohort #1 and 12 in Validation Cohort #2—a total of 36 high‐expressers. Eleven (31%) of these 36 high‐expressers of *TIMD4* had early relapses.

## 4 DISCUSSION

Previous studies reported associations between *ETV6*, *CDKN1B*, *CDKN2A/B*, *CCNC*, *BCL2L14* and *NR3C1* deletions and relapse [11, 12, 15, 17, 18]. They, however, focused on identifying molecular signatures of relapse *after* relapse has already been detected. In contrast, we wanted to identify high‐risk molecular signatures that are already present *at diagnosis*. Leukaemia clones in early relapse usually originate from sub‐clones that are already present at diagnosis, with new mutations being accumulated after treatment [20, 40, 41]. The presence of these sub‐clones at diagnosis indicates the underlying cause of early relapse is a very early event that is possibly amenable to early detection, perhaps as early as at diagnosis. Assessment of early relapse risk at the time of disease presentation might also guide treatment decisions for preventing early relapse [42, 43].

Multi‐omics analyses could discover clinically relevant prognostic factors that might escape single‐omics analysis [44, 45, 46]. In the present study, we conducted a multi‐omics analysis of 87 children with low‐risk *ETV6::RUNX1*‐positive ALL from China. We identified *TIMD4* over‐expression at diagnosis as a prognostic factor for early relapse risk and verified its validity using 153 low‐risk subjects (68 children with *ETV6::RUNX1* and 85 without *ETV6::RUNX1*) from the US.

*TIMD4* (*T‐cell immunoglobulin and mucin domain‐containing molecule 4*) is a cell‐surface glycoprotein in the *TIM* family of genes [47]. In normal subjects, *TIMD4* is exclusively expressed in antigen‐presenting cells such as macrophages and is an important mediator of immune tolerance [47]. Nevertheless, in diseased states, *TIMD4* expression might not be limited to antigen‐presenting cells. In fact, abnormal expression of *TIMD4* has been reported in diverse cancer cells including diffuse large B‐cell lymphoma (DLBCL), non‐small‐cell lung cancer, glioma, renal cell carcinoma and histiocytic sarcoma and usually associated with adverse prognosis [48, 49, 50, 51, 52, 53]. Over‐expression of *TIMD4* in DLBCL cancer cells, lung cancer cells and ovarian cancer‐associated macrophages could promote cancer cell growth through mechanisms such as the Wnt/beta‐catenin pathway and the oxidative phosphorylation pathway [50, 52–54]. *TIMD4* expression in tumour‐associated myeloid cells such as macrophages and dendritic cells promotes autophagy of dying cancer cells and leads to attenuated antigen presentation and increased immune tolerance [55]. Recently, *TIMD4* blockade has been proposed as a potential strategy to enhance the efficacy of CD8^+^ T cell‐based immunotherapies in cancers such as melanoma and lung cancer [56, 57]. Nonetheless, the molecular mechanisms underlying *TIMD4* in leukaemias remain largely unknown. Although in each sample of Discovery Cohort at least half of the mononuclear cells were blasts (presumably leukaemia cells), we cannot be certain whether *TIMD4* over‐expression occurred in the leukaemia cells or tumour‐associated myeloid cells.

Gene expression profiling is becoming ever more important in the diagnosis and prognosis of leukaemia [58, 59, 60, 61, 62]. Our results suggest testing for high *TIMD4* expression in children with low‐risk *ETV6::RUNX1*‐positive B‐cell ALL, and plausibly also in low‐risk *ETV6::RUNX1*‐negative cases, identifies those at high risk for early relapse. Changing therapy in these children might decrease early relapse risk if such a change is proven effective.

Our study has limitations. First, the number of early‐relapse cases in the discovery cohort was small, and this increased the risk of false discovery in multiple‐variable regression analyses. Second, the relapse rate of *ETV6::RUNX1*‐positive subjects in our study was higher than the numbers reported in DCOG ALL10 and NOPHO ALL2008, and this discrepancy in CIR might raise concerns regarding the generalisability of our results [63, 64]. As we described above, one plausible explanation for our discovery cohort's apparently higher CIR is that physicians selectively preserved bone marrow samples of subjects they deemed higher‐ risk, but this cannot be verified. Third, although we did perform cross‐validation of our findings, we could only confirm our results in validation‐cohort subjects whose bone marrow samples had been sequenced, but the physicians’ decision to sequence these patients might have attendant biases. Fourth, more validation effort is needed on additional subjects treated on a broader set of protocols (beyond CCCG‐ALL‐2015, COG AALL0232, COG AALL0331 and Total Therapy 16); until then, we cannot conclude *TIMD4* expression level is a useful biomarker for prognosis in low‐risk childhood ALL. Fifth, our gene expression analyses were done on bulk populations of mononuclear cells isolated from bone marrow samples, and we do not know whether *TIMD4* over‐expression occurred in the leukaemia cells or other cells in the bone marrow. Finally, we did not conduct functional studies on the *TIMD4* gene, and its role in early relapse remains unclear.

## AUTHOR CONTRIBUTIONS

Junren Chen and Xiaofan Zhu conceived the study. Xueou Liu and Qiujin Shen compiled and curated the data, assisted by Yu Hu, Wen Yan, Tiantian Wang, Junxia Wang and Zhen Song. Chengwen Li and Jiao Ma performed assays to confirm the presence/absence of *ETV6::RUNX1*. Xueou Liu, Junren Chen and Xiaofan Zhu planned the re‐sequencing experiments. Tianyuan Hu, Luyang Zhang, Xiaoli Chen, Jiarui Zheng and Aoli Zhang performed the experiments, assisted by Yingchi Zhang. Xiaowen Gong, Wei Zhang, Tiantian Wang, Wen Yan, Xueou Liu, Qiujin Shen and Xiaoyun Li did the analysis, assisted by Robert Peter Gale, Yahui Feng and Xin Gao. Junren Chen and Robert Peter Gale prepared the typescript, assisted by Xiaowen Gong, Wei Zhang, Tiantian Wang, Wen Yan, Qiujin Shen, Yu Hu and Yahui Feng. All authors reviewed the typescript, took responsibility for the content and agreed to submit it for publication.

## CONFLICT OF INTEREST STATEMENT

Robert Peter Gale is a consultant to Antengene Biotech LLC; Medical Director, FFF Enterprises Inc.; A speaker for Janssen Pharma and Hengrui Pharma; Board of Directors: Russian Foundation for Cancer Research Support and Scientific Advisory Board, StemRad Ltd.

## ETHICS STATEMENT

This study was approved by the Academic Committee (IIT‐NI2020001) and Ethics Review Committee (NI2020001‐EC‐1) of the Institute of Hematology, Chinese Academy of Medical Sciences (IHCAMS).

## PATIENT CONSENT STATEMENT

Subjects and/or guardians gave written informed consent, consistent with precepts of the Helsinki Declaration.

## CLINICAL TRIAL REGISTRATION

The authors have confirmed clinical trial registration is not needed for this submission.

## Supporting information

Supporting Information

## Data Availability

Somatic point mutation, gene expression and SNP data have been deposited at the National Genomics Data Center of China with accession codes HRA006311 and OMIX005440. Additional data are available upon reasonable request and addressed to the corresponding authors.
