# Supplementary material for: Gene expression prognostic of early relapse risk in low‐risk B‐cell acute lymphoblastic leukaemia in children
Source: EJHaem. 2024 Mar 15;5(2):333–45. doi: 10.1002/jha2.872 (PMC11020147; doi:10.1002/jha2.872)
Supplement: Supplementary file 1 — Supporting Information [file JHA2-5-333-s001.docx]

Supplement data

Gene expression prognostic of early relapse risk in low-risk B-cell acute lymphoblastic leukaemia in children

**Supplement Table 1.** 559 sequenced genes in Discovery Cohort.

| *ABCB1* | *ABCB11* | *ABCC2* | *ABCC4* | *ABL1* | *ABL2* | *ACSL3* | *ACVR1B* | *ACVR2A* | *ADAMTS20* |
| --- | --- | --- | --- | --- | --- | --- | --- | --- | --- |
| *ADGRA2* | *ADGRB3* | *ADGRL3* | *AFF1* | *AFF3* | *AKAP9* | *AKT1* | *AKT2* | *AKT3* | *ALK* |
| *AMER1* | *APC* | *AR* | *ARID1A* | *ARID1B* | *ARID2* | *ARNT* | *ASXL1* | *ATF1* | *ATM* |
| *ATR* | *ATRX* | *AURKA* | *AURKB* | *AURKC* | *AXIN1* | *AXIN2* | *AXL* | *B2M* | *BAP1* |
| *BCL10* | *BCL11A* | *BCL11B* | *BCL2* | *BCL2L1* | *BCL2L2* | *BCL3* | *BCL6* | *BCL9* | *BCOR* |
| *BCR* | *BIRC2* | *BIRC3* | *BIRC5* | *BLM* | *BLNK* | *BMPR1A* | *BRAF* | *BRCA1* | *BRCA2* |
| *BRD3* | *BRIP1* | *BTK* | *BUB1B* | *C2orf44* | *C8orf34* | *CANT1* | *CARD11* | *CASC5* | *CASP8* |
| *CBL* | *CBR3* | *CCDC6* | *CCND1* | *CCND2* | *CCNE1* | *CD74* | *CD79A* | *CD79B* | *CDA* |
| *CDC73* | *CDH1* | *CDH11* | *CDH2* | *CDH20* | *CDH5* | *CDK12* | *CDK4* | *CDK6* | *CDK8* |
| *CDKN1B* | *CDKN2A* | *CDKN2B* | *CDKN2C* | *CEBPA* | *CHD7* | *CHEK1* | *CHEK2* | *CHIC2* | *CIC* |
| *CKS1B* | *CMPK1* | *COL1A1* | *COL7A1* | *CRBN* | *CREB1* | *CREBBP* | *CRKL* | *CRLF2* | *CRTC1* |
| *CSF1R* | *CSF3R* | *CSMD3* | *CTNNA1* | *CTNNB1* | *CUL4A* | *CUX1* | *CYLD* | *CYP19A1* | *CYP2A6* |
| *CYP2B6* | *CYP2C19* | *CYP2C9* | *CYP2D6* | *DAXX* | *DCC* | *DDB2* | *DDIT3* | *DDR1* | *DDR2* |
| *DDX3X* | *DEK* | *DHFR* | *DICER1* | *DIS3L2* | *DKC1* | *DNMT1* | *DNMT3A* | *DOCK8* | *DPYD* |
| *DST* | *EGFR* | *EIF3E* | *ELANE* | *EML4* | *EP300* | *EP400* | *EPHA3* | *EPHA7* | *EPHB1* |
| *EPHB4* | *EPHB6* | *ERBB2* | *ERBB3* | *ERBB4* | *ERCC1* | *ERCC2* | *ERCC3* | *ERCC4* | *ERCC5* |
| *ERG* | *ESR1* | *ESR2* | *ETS1* | *ETV1* | *ETV4* | *ETV5* | *ETV6* | *EWSR1* | *EXT1* |
| *EXT2* | *EZH2* | *EZR* | *FAH* | *FAM46C* | *FANCA* | *FANCC* | *FANCD2* | *FANCF* | *FANCG* |
| *FAS* | *FBXO7* | *FBXW7* | *FCGR3A* | *FGFR1* | *FGFR2* | *FGFR3* | *FGFR4* | *FH* | *FLCN* |
| *FLI1* | *FLT1* | *FLT3* | *FLT4* | *FN1* | *FOXA1* | *FOXL2* | *FOXO1* | *FOXO3* | *FOXO4* |
| *FOXP1* | *FOXP4* | *FSTL5* | *FUBP1* | *FZR1* | *G6PD* | *GATA1* | *GATA2* | *GATA3* | *GBA* |
| *GDNF* | *GJB2* | *GNA11* | *GNAQ* | *GNAS* | *GPC3* | *GRIN2A* | *GRM8* | *GSTP1* | *GUCY1A2* |
| *GUCY2F* | *H3F3A* | *HCAR1* | *HFE* | *HIF1A* | *HIST1H3B* | *HLF* | *HMBS* | *HNF1A* | *HOOK3* |
| *HRAS* | *HSP90AA1* | *HSP90AB1* | *IDH1* | *IDH2* | *IGF1R* | *IGF2* | *IGF2R* | *IKBKB* | *IKBKE* |
| *IKZF1* | *IL2* | *IL21R* | *IL2RA* | *IL2RB* | *IL2RG* | *IL6ST* | *IL7R* | *ING4* | *INPP4B* |
| *IRF4* | *IRS2* | *ITGA10* | *ITGA9* | *ITGB2* | *ITGB3* | *ITK* | *JAK1* | *JAK2* | *JAK3* |
| *JUN* | *KAT6A* | *KAT6B* | *KDM5C* | *KDM6A* | *KDR* | *KEAP1* | *KIF5B* | *KIT* | *KLF4* |
| *KLF6* | *KMT2A* | *KMT2C* | *KMT2D* | *KRAS* | *LAMA2* | *LAMP1* | *LCK* | *LIFR* | *LPP* |
| *LRIG3* | *LRP1B* | *LTF* | *LTK* | *MAF* | *MAFB* | *MAGEA1* | *MAGI1* | *MALT1* | *MAML2* |
| *MAP2K1* | *MAP2K2* | *MAP2K4* | *MAP3K1* | *MAP3K7* | *MAPK1* | *MAPK8* | *MARK1* | *MARK4* | *MAX* |
| *MBD1* | *MCL1* | *MDM2* | *MDM4* | *MED12* | *MED13* | *MEN1* | *MET* | *MITF* | *MLH1* |
| *MLLT10* | *MMP2* | *MN1* | *MPL* | *MRE11A* | *MSH2* | *MSH6* | *MST1R* | *MTAP* | *MTHFR* |
| *MTOR* | *MTR* | *MTRR* | *MUC1* | *MUTYH* | *MYB* | *MYC* | *MYCL* | *MYCN* | *MYD88* |
| *MYH11* | *MYH9* | *NBN* | *NCOA1* | *NCOA2* | *NCOA4* | *NCOR1* | *NELL2* | *NF1* | *NF2* |
| *NFE2L2* | *NFKB1* | *NFKB2* | *NIN* | *NKX2-1* | *NLRP1* | *NME1* | *NOTCH1* | *NOTCH2* | *NOTCH4* |
| *NPM1* | *NRAS* | *NRG1* | *NSD1* | *NTRK1* | *NTRK3* | *NUMA1* | *NUP214* | *NUP98* | *PAK3* |
| *PALB2* | *PARP1* | *PAX3* | *PAX5* | *PAX7* | *PAX8* | *PBRM1* | *PBX1* | *PDE4DIP* | *PDGFB* |
| *PDGFRA* | *PDGFRB* | *PER1* | *PGAP3* | *PHF6* | *PHOX2B* | *PIK3C2B* | *PIK3CA* | *PIK3CB* | *PIK3CD* |
| *PIK3CG* | *PIK3R1* | *PIK3R2* | *PIM1* | *PKHD1* | *PLAG1* | *PLCG1* | *PLEKHG5* | *PML* | *PMS1* |
| *PMS2* | *POLD1* | *POLE* | *POLH* | *POT1* | *POU5F1* | *PPARG* | *PPP2R1A* | *PRDM1* | *PRF1* |
| *PRKAR1A* | *PRKDC* | *PRSS1* | *PSIP1* | *PSMB1* | *PSMB2* | *PSMB5* | *PSMD1* | *PSMD2* | *PTCH1* |
| *PTEN* | *PTGS2* | *PTPN11* | *PTPRD* | *PTPRK* | *PTPRT* | *RAC1* | *RAD21* | *RAD50* | *RAD51C* |
| *RAD51D* | *RAF1* | *RALGDS* | *RARA* | *RARB* | *RARG* | *RB1* | *RECQL4* | *REL* | *RET* |
| *RHBDF2* | *RHOH* | *RNASEL* | *RNF2* | *RNF213* | *RNF43* | *ROS1* | *RPS6KA2* | *RPS6KB1* | *RRM1* |
| *RSPO2* | *RSPO3* | *RUNX1* | *RUNX1T1* | *RXRA* | *RXRB* | *RXRG* | *SAMD9* | *SBDS* | *SDC4* |
| *SDHA* | *SDHAF2* | *SDHB* | *SDHC* | *SDHD* | *SEPT9* | *SERPINA1* | *SETBP1* | *SETD2* | *SF3B1* |
| *SGK1* | *SH2B3* | *SH2D1A* | *SHH* | *SHOC2* | *SLC22A1* | *SLC22A2* | *SLC25A13* | *SLC31A1* | *SLC34A2* |
| *SLC45A3* | *SLCO1B1* | *SMAD2* | *SMAD4* | *SMARCA4* | *SMARCB1* | *SMARCD1* | *SMARCE1* | *SMO* | *SMUG1* |
| *SNCAIP* | *SOCS1* | *SOD2* | *SOS1* | *SOX11* | *SOX2* | *SOX9* | *SPOP* | *SPRED1* | *SRC* |
| *SRSF2* | *SSX1* | *STAG2* | *STAT3* | *STK11* | *STK36* | *SUFU* | *SYK* | *SYNE1* | *TAF1* |
| *TAF1L* | *TAL1* | *TAS2R38* | *TBX22* | *TCF12* | *TCF3* | *TCF7L1* | *TCF7L2* | *TCL1A* | *TERT* |
| *TET1* | *TET2* | *TFE3* | *TGFBR1* | *TGFBR2* | *TGM7* | *THBS1* | *TIMP3* | *TLR4* | *TLX1* |
| *TMEM127* | *TNFAIP3* | *TNFRSF14* | *TNK2* | *TOP1* | *TP53* | *TPMT* | *TPR* | *TRAF7* | *TRIM24* |
| *TRIM33* | *TRIM37* | *TRIP11* | *TRRAP* | *TSC1* | *TSC2* | *TSHR* | *TYK2* | *U2AF1* | *UBR5* |
| *UGT1A1* | *UGT1A9* | *UMPS* | *UROD* | *USP9X* | *VHL* | *WAS* | *WHSC1* | *WRN* | *WT1* |
| *XPA* | *XPC* | *XPO1* | *XRCC1* | *XRCC2* | *YES1* | *ZMYM3* | *ZNF384* | *ZNF521* |  |

**Supplement Table 2.** Clinical characteristics of low-risk *ETV6::RUNX1*-positive childhood ALL patients with preserved bone marrow samples and those without at the IHCAMS.

|  | **Patients with preserved bone marrow samples**  **(N = 110)** | **Patients without preserved bone marrow samples**  **(N = 98)** | ***P*-value** |
| --- | --- | --- | --- |
| **Age at diagnosis, median years (range)** | 4.0 (1.4 – 10.0) | 4.9 (1.3 – 9.8) | 0.10 ^*^ |
| **Biological sex, N (%)** |  |  | 0.22 ^†^ |
| Male | 57 (51.8) | 60 (61.2) |  |
| Female | 53 (48.2) | 38 (38.8) |  |
| **Ethnicity, N (%)**  Asian | 110 (100.0) | 98 (100) | – |
| **WBC count at diagnosis, median**  **× 10E+9/L (range)** | 6.9 (0.9 – 93.1) | 7.9 (1.3 – 127.3) | 0.58 ^*^ |
| **Disease risk at diagnosis, N (%)** ^a^  Low-risk | 110 (100.0) | 98 (100) | – |
| **Treatment regimen, N (%)**  CCCG-ALL-2015 | 110 (100.0) | 98 (100) | – |
| **MRD > 1 × 10E–2 on day 19, N (%)** | 11 (10.0) | 6 (6.1) | 0.48 ^†^ |
| **MRD > 0 on day 46, N (%)** | 23 (20.9) | 13 (13.3) | 0.22 ^†^ |
| **Follow-up duration, median years (IQR)** | 4.1 (3.0 – 5.7) | 5.3 (3.4 – 6.2) | 0.006 ^§^ |
| **Relapse rate, % (95% CI)** |  |  | 0.16 ^‡^ |
| 3 years | 9.2 (4.5, 16.0) | 3.5 (0.9, 9.2) |  |
| 5 years | 15.2 (7.9, 24.8) | 7.9 (3.1, 15.5) |  |
| **Survival, % (95% CI)** |  |  | 0.70 ^§^ |
| 3 years | 97.8 (94.8, 100.0) | 97.9 (95.1, 100.0) |  |
| 5 years | 95.7 (90.9, 100.0) | 97.9 (95.1, 100.0) |  |

Abbreviations: ALL, acute lymphoblastic leukemia; CI, confidence interval; IQR, interquartile range; MRD, measurable residual disease; WBC, white blood cell.

^a^ Risk-stratification was based on subject characteristics *at diagnosis* according to CCCG-ALL-2015 criteria.

^*^ Two-tailed Wilcoxon test

^†^ Chi-squared test

^‡^ Gray test

^§^ Two-tailed log-rank test

**Supplement Table 3.** Subjects in Validation Cohort #1 from the US (N = 68).

| **Sample ID** | **Type** | **Risk at**  **diagnosis ^a^** | **Age at**  **diagnosis**  **(years)** | **WBC at diagnosis**  **(× 10E+9/L)** | **Sex** | **Race** | **Protocol** | **Early relapse** | **Early relapse**  **time (years)** |
| --- | --- | --- | --- | --- | --- | --- | --- | --- | --- |
| SJBALL020627 | B Cell | Low | 4.5 | 73.6 | Female | Black or African American | AALL0232 | 0 | 0.9 |
| SJCOGALL010896 | B Cell | Low | 1.9 | 77.9 | Male | White | AALL0232 | 1 | 2.1 |
| SJBALL021187 | B Cell | Low | 3.1 | 12.5 | Male | Unknown | AALL0331 | 1 | 0.9 |
| SJCOGALL010229 | B Cell | Low | 8.4 | 28.3 | Female | White | AALL0331 | 1 | 3.0 |
| SJBALL020690 | B Cell | Low | 5.2 | 66.8 | Female | White | AALL0232 | 0 | – |
| SJBALL020630 | B Cell | Low | 3.5 | 63.4 | Male | Unknown | AALL0232 | 0 | – |
| SJCOGALL010869 | B Cell | Low | 6.9 | 20.5 | Female | Unknown | AALL0331 | 1 | 1.8 |
| SJBALL021234 | B Cell | Low | 3.6 | 12.9 | Male | White | AALL0331 | 0 | – |
| SJBALL020684 | B Cell | Low | 6.0 | 117.6 | Female | White | AALL0232 | 0 | – |
| SJCOGALL010917 | B Cell | Low | 2.8 | 47.7 | Female | White | AALL0331 | 1 | 2.8 |
| SJCOGALL010885 | B Cell | Low | 4.0 | 3.5 | Male | Black or African American | AALL0331 | 1 | 2.2 |
| SJBALL020593 | B Cell | Low | 6.7 | 55.2 | Male | White | AALL0232 | 0 | – |
| SJBALL020660 | B Cell | Low | 4.3 | 85.0 | Male | White | AALL0232 | 0 | – |
| SJBALL020583 | B Cell | Low | 2.3 | 106.5 | Male | White | AALL0232 | 0 | – |
| SJBALL020487 | B Cell | Low | 2.4 | 51.6 | Male | White | AALL0232 | 1 | 2.5 |
| SJBALL020604 | B Cell | Low | 6.7 | 81.6 | Female | White | AALL0232 | 0 | – |
| SJBALL020728 | B Cell | Low | 2.9 | 82.2 | Female | White | AALL0232 | 0 | – |
| SJCOGALL010862 | B Cell | Low | 5.6 | 20.3 | Male | White | AALL0331 | 0 | – |
| SJBALL020653 | B Cell | Low | 6.9 | 90.8 | Male | White | AALL0232 | 0 | – |
| SJBALL020610 | B Cell | Low | 3.4 | 63.0 | Male | White | AALL0232 | 0 | – |
| SJBALL020662 | B Cell | Low | 2.5 | 79.3 | Male | White | AALL0232 | 0 | – |
| SJALL040128 | B Cell | Low | 5.0 | 3.1 | Male | White | Total Therapy 16 | 0 | – |
| SJETV021993 | B Cell | Low | 4.2 | 5.2 | Female | White | Total Therapy 16 | 0 | – |
| SJETV021986 | B Cell | Low | 4.6 | 6.2 | Male | White | Total Therapy 16 | 0 | – |
| SJETV022049 | B Cell | Low | 5.2 | 11.1 | Female | Black | Total Therapy 16 | 0 | – |
| SJETV022010 | B Cell | Low | 8.9 | 12.5 | Male | White | Total Therapy 16 | 0 | – |
| SJALL040135 | B Cell | Low | 1.9 | 22.7 | Male | Other | Total Therapy 16 | 0 | – |
| SJETV021949 | B Cell | Low | 9.9 | 35.7 | Female | White | Total Therapy 16 | 0 | – |
| SJETV021952 | B Cell | Low | 5.1 | 23.6 | Male | White | Total Therapy 16 | 0 | – |
| SJETV021963 | B Cell | Low | 3.3 | 48.3 | Male | White | Total Therapy 16 | 0 | – |
| SJALL040063 | B Cell | Low | 3.2 | 22.9 | Female | White | Total Therapy 16 | 0 | – |
| SJALL040093 | B Cell | Low | 2.9 | 16.3 | Female | Other | Total Therapy 16 | 0 | – |
| SJETV021939 | B Cell | Low | 2.9 | 9.9 | Male | White | Total Therapy 16 | 0 | – |
| SJETV021945 | B Cell | Low | 4.4 | 4.3 | Male | White | Total Therapy 16 | 0 | – |
| SJETV022015 | B Cell | Low | 3.9 | 15.4 | Male | White | Total Therapy 16 | 0 | – |
| SJETV021956 | B Cell | Low | 3.8 | 42.2 | Female | Black | Total Therapy 16 | 0 | – |
| SJETV022026 | B Cell | Low | 4.3 | 31.8 | Female | White | Total Therapy 16 | 0 | 0.1 |
| SJALL040059 | B Cell | Low | 5.5 | 2.1 | Male | White | Total Therapy 16 | 0 | – |
| SJETV021967 | B Cell | Low | 2.6 | 20.6 | Male | White | Total Therapy 16 | 0 | – |
| SJETV021969 | B Cell | Low | 9.0 | 4.5 | Male | White | Total Therapy 16 | 0 | – |
| SJALL040061 | B Cell | Low | 4.8 | 15.0 | Male | White | Total Therapy 16 | 0 | – |
| SJALL040117 | B Cell | Low | 5.1 | 52.0 | Female | White | Total Therapy 16 | 0 | – |
| SJETV021937 | B Cell | Low | 3.8 | 30.9 | Male | White | Total Therapy 16 | 0 | – |
| SJETV021953 | B Cell | Low | 2.5 | 33.0 | Female | White | Total Therapy 16 | 0 | – |
| SJALL040077 | B Cell | Low | 2.3 | 17.5 | Female | White | Total Therapy 16 | 0 | – |
| SJALL040081 | B Cell | Low | 3.2 | 66.5 | Male | White | Total Therapy 16 | 0 | – |
| SJETV021977 | B Cell | Low | 4.2 | 6.6 | Male | Black | Total Therapy 16 | 0 | – |
| SJALL040090 | B Cell | Low | 3.3 | 26.7 | Male | Black | Total Therapy 16 | 0 | – |
| SJETV021948 | B Cell | Low | 3.1 | 2.9 | Male | White | Total Therapy 16 | 0 | – |
| SJALL040106 | B Cell | Low | 4.3 | 7.1 | Female | Black | Total Therapy 16 | 0 | – |
| SJETV022023 | B Cell | Low | 3.1 | 31.7 | Male | White | Total Therapy 16 | 0 | – |
| SJETV022019 | B Cell | Low | 3.4 | 6.7 | Male | White | Total Therapy 16 | 0 | – |
| SJETV021980 | B Cell | Low | 2.9 | 19.9 | Male | Black | Total Therapy 16 | 0 | – |
| SJALL040068 | B Cell | Low | 1.7 | 64.5 | Male | White | Total Therapy 16 | 0 | – |
| SJETV021927 | B Cell | Low | 2.3 | 72.4 | Female | Black | Total Therapy 16 | 0 | – |
| SJALL040067 | B Cell | Low | 4.0 | 13.0 | Male | White | Total Therapy 16 | 0 | – |
| SJALL040086 | B Cell | Low | 2.6 | 77.4 | Female | White | Total Therapy 16 | 0 | – |
| SJETV021997 | B Cell | Low | 6.1 | 40.4 | Female | White | Total Therapy 16 | 0 | – |
| SJETV021942 | B Cell | Low | 4.1 | 24.3 | Female | White | Total Therapy 16 | 0 | – |
| SJETV021990 | B Cell | Low | 2.1 | 23.2 | Male | White | Total Therapy 16 | 0 | – |
| SJALL040082 | B Cell | Low | 7.3 | 7.3 | Male | White | Total Therapy 16 | 0 | – |
| SJALL040104 | B Cell | Low | 3.6 | 23.9 | Female | Black | Total Therapy 16 | 0 | – |
| SJETV021981 | B Cell | Low | 8.1 | 111.1 | Female | Black | Total Therapy 16 | 0 | – |
| SJETV021959 | B Cell | Low | 2.9 | 18.6 | Female | White | Total Therapy 16 | 0 | – |
| SJALL040084 | B Cell | Low | 2.3 | 51.5 | Male | White | Total Therapy 16 | 0 | – |
| SJALL040123 | B Cell | Low | 8.0 | 16.6 | Female | White | Total Therapy 16 | 0 | – |
| SJETV021972 | B Cell | Low | 3.0 | 10.2 | Female | Black | Total Therapy 16 | 0 | – |
| SJALL040118 | B Cell | Low | 2.2 | 10.4 | Male | White | Total Therapy 16 | 0 | – |

^a^ Risk-stratification was according to the CCCG-ALL-2015 criteria.

**Supplement Table 4.** Subjects in Validation Cohort #2 from the US (N = 78).

| **Sample ID** | **Type** | **Risk at diagnosis ^a^** | **Age at  diagnosis (years)** | **WBC at diagnosis (× 10E+9/L)** | **Sex** | **Race** | **Protocol** | **Early relapse** | **Early relapse time (years)** |
| --- | --- | --- | --- | --- | --- | --- | --- | --- | --- |
| SJCOGALL010859 | B Cell | Low | 7.6 | 21.8 | Female | Unknown | AALL0331 | 0 | – |
| SJCOGALL010860 | B Cell | Low | 2.8 | 6.4 | Male | White | AALL0331 | 1 | 2.8 |
| SJBALL021240 | B Cell | Low | 6.5 | 15.2 | Female | White | AALL0331 | 1 | 2.6 |
| SJCOGALL010221 | B Cell | Low | 2.3 | 6.0 | Female | Black or African American | AALL0331 | 1 | 2.1 |
| SJBALL021188 | B Cell | Low | 9.6 | 5.0 | Female | White | AALL0331 | 1 | 2.4 |
| SJBALL021197 | B Cell | Low | 1.4 | 44.0 | Male | Unknown | AALL0331 | 1 | 2.1 |
| SJBALL021237 | B Cell | Low | 4.9 | 12.1 | Male | White | AALL0331 | 0 | – |
| SJCOGALL010222 | B Cell | Low | 7.0 | 18.0 | Female | White | AALL0331 | 1 | 2.2 |
| SJCOGALL010870 | B Cell | Low | 3.7 | 7.7 | Female | White | AALL0331 | 0 | – |
| SJCOGALL010919 | B Cell | Low | 2.3 | 18.5 | Male | White | AALL0331 | 0 | – |
| SJCOGALL010918 | B Cell | Low | 8.9 | 2.3 | Female | White | AALL0331 | 0 | – |
| SJBALL021159 | B Cell | Low | 7.3 | 16.2 | Female | Unknown | AALL0331 | 1 | 1.5 |
| SJBALL021235 | B Cell | Low | 2.9 | 9.9 | Male | Unknown | AALL0331 | 0 | – |
| SJBALL021239 | B Cell | Low | 4.1 | 8.1 | Female | White | AALL0331 | 0 | – |
| SJBALL021264 | B Cell | Low | 2.2 | 23.5 | Male | White | AALL0331 | 1 | 2.2 |
| SJCOGALL010220 | B Cell | Low | 7.1 | 22.2 | Male | White | AALL0331 | 1 | 3.0 |
| SJCOGALL010234 | B Cell | Low | 4.2 | 21.5 | Male | White | AALL0331 | 1 | 2.2 |
| SJCOGALL010889 | B Cell | Low | 3.0 | 8.4 | Female | White | AALL0331 | 1 | 0.6 |
| SJCOGALL010907 | B Cell | Low | 5.4 | 31.2 | Female | Asian | AALL0331 | 0 | – |
| SJCOGALL011124 | B Cell | Low | 5.5 | 13.7 | Female | White | AALL0331 | 0 | – |
| SJCOGALL010871 | B Cell | Low | 5.1 | 15.3 | Male | White | AALL0331 | 1 | 1.6 |
| SJBALL021248 | B Cell | Low | 2.9 | 7.1 | Female | White | AALL0331 | 0 | – |
| SJCOGALL010223 | B Cell | Low | 2.5 | 1.3 | Female | White | AALL0331 | 1 | 2.3 |
| SJCOGALL010890 | B Cell | Low | 3.1 | 2.4 | Male | White | AALL0331 | 0 | – |
| SJCOGALL010908 | B Cell | Low | 2.8 | 21.9 | Male | White | AALL0331 | 1 | 2.7 |
| SJBALL021245 | B Cell | Low | 3.3 | 5.6 | Male | White | AALL0331 | 0 | – |
| SJBALL224 | B Cell | Low | 2.5 | 18.6 | Female | Black | Total Therapy 16 | 0 | – |
| SJBALL218 | B Cell | Low | 7.5 | 38.3 | Male | White | Total Therapy 16 | 0 | – |
| SJBALL021995 | B Cell | Low | 1.7 | 43.6 | Male | Other | Total Therapy 16 | 0 | – |
| SJBALL021941 | B Cell | Low | 7.9 | 8.4 | Male | White | Total Therapy 16 | 0 | – |
| SJBALL022020 | B Cell | Low | 2.7 | 38.7 | Female | Other | Total Therapy 16 | 0 | – |
| SJBALL021960 | B Cell | Low | 1.9 | 14.1 | Male | White | Total Therapy 16 | 0 | – |
| SJHYPO022024 | B Cell | Low | 1.8 | 11.2 | Female | Other | Total Therapy 16 | 0 | – |
| SJBALL247 | B Cell | Low | 1.6 | 7.5 | Male | White | Total Therapy 16 | 0 | – |
| SJALL040053 | B Cell | Low | 4.2 | 5.2 | Male | White | Total Therapy 16 | 0 | – |
| SJALL040060 | B Cell | Low | 2.2 | 13.0 | Male | White | Total Therapy 16 | 0 | – |
| SJALL040064 | B Cell | Low | 2.0 | 49.9 | Female | White | Total Therapy 16 | 0 | – |
| SJALL040076 | B Cell | Low | 3.5 | 25.0 | Male | White | Total Therapy 16 | 0 | – |
| SJALL040080 | B Cell | Low | 8.3 | 7.2 | Male | White | Total Therapy 16 | 0 | – |
| SJALL040091 | B Cell | Low | 7.5 | 3.5 | Female | White | Total Therapy 16 | 0 | – |
| SJALL040099 | B Cell | Low | 9.8 | 3.7 | Male | White | Total Therapy 16 | 0 | – |
| SJALL040111 | B Cell | Low | 1.8 | 26.2 | Female | White | Total Therapy 16 | 0 | – |
| SJALL040114 | B Cell | Low | 6.6 | 15.6 | Female | White | Total Therapy 16 | 0 | – |
| SJALL040115 | B Cell | Low | 3.7 | 8.0 | Female | White | Total Therapy 16 | 0 | – |
| SJALL040120 | B Cell | Low | 6.3 | 9.5 | Male | Black | Total Therapy 16 | 0 | – |
| SJALL040122 | B Cell | Low | 7.8 | 13.2 | Male | White | Total Therapy 16 | 0 | – |
| SJALL040132 | B Cell | Low | 6.8 | 32.9 | Female | White | Total Therapy 16 | 0 | – |
| SJHYPER021984 | B Cell | Low | 3.4 | 15.6 | Female | White | Total Therapy 16 | 0 | – |
| SJHYPER206 | B Cell | Low | 7.1 | 5.4 | Female | White | Total Therapy 16 | 0 | – |
| SJHYPER021996 | B Cell | Low | 4.1 | 6.4 | Male | White | Total Therapy 16 | 0 | – |
| SJHYPER021925 | B Cell | Low | 5.3 | 13.0 | Female | White | Total Therapy 16 | 0 | – |
| SJHYPER022003 | B Cell | Low | 6.7 | 45.2 | Female | White | Total Therapy 16 | 0 | – |
| SJHYPER021934 | B Cell | Low | 2.9 | 7.0 | Female | White | Total Therapy 16 | 0 | – |
| SJHYPER021935 | B Cell | Low | 5.4 | 2.1 | Female | White | Total Therapy 16 | 0 | – |
| SJHYPER022008 | B Cell | Low | 3.1 | 16.7 | Female | White | Total Therapy 16 | 0 | – |
| SJHYPER022013 | B Cell | Low | 5.3 | 0.2 | Male | White | Total Therapy 16 | 0 | – |
| SJHYPER022016 | B Cell | Low | 3.2 | 7.0 | Male | White | Total Therapy 16 | 0 | – |
| SJHYPER022017 | B Cell | Low | 4.2 | 33.6 | Male | White | Total Therapy 16 | 0 | – |
| SJHYPER021946 | B Cell | Low | 5.3 | 4.3 | Male | White | Total Therapy 16 | 0 | – |
| SJHYPER227 | B Cell | Low | 2.3 | 7.0 | Male | Black | Total Therapy 16 | 0 | – |
| SJHYPER021950 | B Cell | Low | 4.3 | 284.6 | Male | White | Total Therapy 16 | 0 | – |
| SJHYPER021954 | B Cell | Low | 4.3 | 3.1 | Male | White | Total Therapy 16 | 0 | – |
| SJHYPER021957 | B Cell | Low | 9.0 | 1.3 | Female | White | Total Therapy 16 | 0 | – |
| SJHYPER021961 | B Cell | Low | 7.6 | 6.1 | Male | White | Total Therapy 16 | 0 | – |
| SJHYPER022027 | B Cell | Low | 3.2 | 28.1 | Female | Black | Total Therapy 16 | 0 | – |
| SJHYPER022032 | B Cell | Low | 2.5 | 15.0 | Male | White | Total Therapy 16 | 0 | – |
| SJHYPER022034 | B Cell | Low | 3.1 | 1.9 | Female | White | Total Therapy 16 | 0 | – |
| SJHYPER021970 | B Cell | Low | 3.5 | 1.3 | Male | White | Total Therapy 16 | 0 | – |
| SJALL040078 | B Cell | Low | 8.0 | 30.3 | Female | Black | Total Therapy 16 | 0 | – |
| SJALL040085 | B Cell | Low | 6.5 | 45.9 | Male | White | Total Therapy 16 | 0 | – |
| SJALL040087 | B Cell | Low | 2.0 | 20.0 | Male | White | Total Therapy 16 | 0 | – |
| SJALL040088 | B Cell | Low | 2.6 | 29.8 | Female | Other | Total Therapy 16 | 0 | – |
| SJALL040102 | B Cell | Low | 1.4 | 5.2 | Female | White | Total Therapy 16 | 0 | – |
| SJALL040116 | B Cell | Low | 3.7 | 31.9 | Female | White | Total Therapy 16 | 0 | – |
| SJALL040124 | B Cell | Low | 3.1 | 27.1 | Female | White | Total Therapy 16 | 0 | – |
| SJALL040126 | B Cell | Low | 9.6 | 2.8 | Male | Black | Total Therapy 16 | 0 | – |
| SJALL040127 | B Cell | Low | 2.0 | 12.3 | Female | White | Total Therapy 16 | 0 | – |
| SJALL040136 | B Cell | Low | 2.6 | 18.8 | Female | White | Total Therapy 16 | 0 | – |

^a^ Risk-stratification was according to the CCCG-ALL-2015 criteria.
